# Evidence for phenotypic plasticity in response to photic cues and the connection with genes of risk in schizophrenia

**DOI:** 10.3389/fnbeh.2013.00082

**Published:** 2013-07-09

**Authors:** Christine L. Miller

**Affiliations:** MillerBioBaltimore, MD, USA

**Keywords:** schizophrenia, pyschoses, epidemiology, photoperiod, natural light, prenatal, melanotropin, vitamin D

## Abstract

Numerous environmental factors have been identified as influential in the development of schizophrenia. Some are byproducts of modern life, yet others were present in our evolutionary past and persist to a lesser degree in the current era. The present study brings together published epidemiological data for schizophrenia and data on variables related to photic input for places of residence across geographical regions, using rainfall as an inverse, proxy measure for light levels. Data were gathered from the literature for two countries, the former Yugoslavia and Ireland, during a time in the early 20th century when mobility was relatively limited. The data for Yugoslavia showed a strong correlation between hospital census rates for schizophrenia (by place of birth) and annual rain (*r* = 0.96, *p* = 0.008). In Ireland, the hospital census rates and first admissions for schizophrenia (by place of permanent residence) showed a trend for correlation with annual rain, reaching significance for 1st admissions when the rainfall data was weighted by the underlying population distribution (*r* = 0.71, *p* = 0.047). In addition, across the years 1921–1945, birth-year variations in a spring quarter season-of-birth effect for schizophrenia in Ireland showed a trend for correlation with January-March rainfall (*r* = 0.80, *p* ≤ 0.10). The data are discussed in terms of the effect of photoperiod on the gestation and behavior of offspring in animals, and the premise is put forth that vestigial phenotypic plasticity for such photic cues still exists in humans. Moreover, genetic polymorphisms of risk identified for psychotic disorders include genes modulated by photoperiod and sunlight intensity. Such a relationship between phenotypic plasticity in response to a particular environmental regime and subsequent natural selection for fixed changes in the environmentally responsive genes, has been well studied in animals and should not be discounted when considering human disease.

## Introduction

The epidemiology of psychiatric disease represents an invaluable resource for new insights into gene-environment interactions as a cause of mental illness. That epidemiological variation in incidence must occur across space and time is consistent with known principles for all human disease, be the cause predominantly environmental or genetic. Although no field of endeavor is so fraught with potentially confounding variables, the perceived difficulties in interpretation should not lead to a blanket rejection of such work. As evidence builds for a consistent trend between studies, and as data mounts from research avenues in genetics and pharmacology that support the epidemiologic results, the resulting knowledge can be used to more productively design future research. Such is the case with three epidemiologic outcomes for schizophrenia that are likely related: the effect of latitude on rates of disease in the indigenous population, the effect of immigration from southern to northern latitudes and the late winter-to-spring quarter season of birth effect, a modest but consistent finding that has survived mathematical challenges (Lewis and Griffin, 1981; Dalén, 1990; Pulver et al., 1990; Watson, 1990), and questions as to its relevance in the Southern hemisphere (McGrath and Welham, 1999), where the effect is much less robust.

One key environmental link between the epidemiological studies and related genetic/pharmacologic results is photic input (McGrath et al., 2002), a factor also of relevance to the melanotropin genes shown to be associated with psychotic disorders including schizophrenia (Severinsen et al., 2006; Miller et al., 2009; Demontis et al., 2012) and of relevance to pharmacological results that pertain to the function of those genes (Miller, 2013). If photic input is the key variable, the less pronounced Southern hemisphere results actually bolster the season-of-birth theory because a much lower percent of populated land mass occurs at the higher latitudes in the southern hemisphere than in the Northern. Importantly, Brisbane, the most populous city in the McGrath et al. study of Australia (2002), rests at latitude 27.5°S. Dublin, in contrast, is at latitude 53.4°N. A meta-analysis of published season of birth studies demonstrated that the effect does go up with increasing latitude (Davies et al., 2003). Furthermore, variation in overall schizophrenia incidence would be expected to vary with latitude, and a meta-analysis by McGrath and colleagues (Saha et al., 2006) demonstrated unequivocally that a gradient exists. This conclusion conflicts with an earlier report sponsored by the World Health Organization (WHO) discounted any correlation with latitude (Jablensky et al., 1992), but their results were somewhat compromised by selective inclusion of one study site in the final report but not another (Chandigarh, an area with a large Sikh population was included, but not Agra, an area with a large Muslim population). Data from their preliminary report (Sartorius et al., 1986) showed a nearly 10-fold difference between the two sites, but the final report (Jablensky et al., 1992) deemed that only suspected methodological differences could explain such geographical variation in incidence on such a small scale despite the fact that differences in racial composition alone could be the basis for those differences. For the study site in Ireland, a country previously reported to have a very high incidence of schizophrenia (Walsh, 1968; Kelleher et al., 1974), the WHO study team selected Dublin as a center for data collection, a region in Ireland not reported to have the high 1st admission rates for schizophrenia more characteristic of the west of Ireland (Kelleher et al., 1974).

When examining the effect of an environmental variable, it is always helpful to first look at the extremes. Nowhere has lack of photic input exerted more effects on humans over time than in the high latitude country of Ireland, where comparatively low dietary vitamin D and the lack of sunlight-induced vitamin D selected for the fairest skin type in the world, as reported in dermatological surveys (Gibson et al., 1997). Yet, although most Swedish residents live at higher latitudes than the Irish, their proportion of skin type 1 and 2 is not as high (Karlsson et al., 2000; Rodvall et al., 2007). Even in the far reaches of populations in the Arctic Circle, the impact of low light on skin type prevalence (Karlsson et al., 2000) was not so extreme, most likely because a diet rich in vitamin D from seafood helped to mitigate the lack of sun. The relationship between a diet rich in fatty fish and serum levels of vitamin D is clear (Burgaz et al., 2007).

McGrath and Welham (1999) have proposed that vitamin D availability may modulate the eventual development of schizophrenia, and Kinney et al. (2009) have extended that theory to propose the risk for schizophrenia around the world is related to levels of vitamin D from fish in their current diet. A complementary hypothesis is that a diet rich in fatty fish actually changed the evolutionary trajectory for some populations. From the time of the Vikings on, the Nordic cultures developed such a robust fishing enterprise that they exported their products to many other European destinations (Sicking and Abreu-Ferreira, 2008). The Irish, in contrast, failed to develop an historically strong sea-faring and fishing industry (Donnchadha et al., 2002), in part because the coastline toward which they were pushed during British occupation (beyond “The Pale”; McManus, 1931) was dangerously rocky and difficult to trawl (Woodham-Smith, 1962). The dire impact of this situation was most apparent during the potato famine, when a marine diet might have saved millions of lives (Donnchadha et al., 2002; Woodham-Smith, 1962). But over the generations, lacking readily available nutrients from the sea meant the resulting deficiency of vitamin D from either diet or the sun selected for an extremely fair skin type, which helped their descendents avoid rickets.

Clearly, the relative lack of sunlight had an evolutionary impact on genes affecting vitamin D generation from sunlight, but what evidence is there that it might also have selected for a change in the prevalence of a disease such as schizophrenia? Might there be vestigial phenotypic plasticity that provides a window into the forces that shaped our evolutionary past? In the animal kingdom, photic cues are crucial to survival, and animals that are adapted to life in regions of low light have evolved to have different light-responsive genotypes than those that evolved near the equator. Yet phenotypic plasticity can also be found, as evidenced by the well-studied effect of photoperiod on gestation, an effect which can not only determine coat color at birth but also neo-natal behavior (Hoffman, 1978; Reppert, 1985; Stetson et al., 1986; Weaver et al., 1987; Lee and Zucker, 1988; Nagy et al., 1993; Bellavía et al., 2006; Butler et al., 2007). Perhaps, then, the season-of-birth effect in schizophrenia could well be evidence of vestigial phenotypic plasticity in response to seasonally varying levels of light.

The approach taken in the present study was to analyze the correlation between rates of schizophrenia and a proxy measure for photic input, rainfall. Two countries were selected for inclusion, one at a more extreme latitude, Ireland, and one at a more moderate latitude, the former Yugoslavia. No attempt was made to compare the two, as a meta-analysis of numerous country-to-country differences has been well performed by others (Saha et al., 2006). Rather, the question being asked was whether small scale geographical differences in prevalence and incidence exist within each country and whether those differences might relate to variations in rainfall. These two countries offered the advantage of weather extremes they encompass, as well as the availability of detailed schizophrenia epidemiology during the early to mid-20th century, at a time when mobility was limited compared to today's world and when the chances were good that someone born in an area would be quite likely to grow up in the same town. The former Yugoslavia represented an opportunity to examine annual rainfall extremes within one country, as its western coastal range home to a region that receives more rainfall than any other in continental Europe, a district in the current Montenegro (Papp and Erzberger, 2007) and the nearby town of Crkvice, Croatia (Krause and Flood, 1997; Marinkovic et al., 2012), while the southern and eastern-most regions of the former country were quite dry. Ireland, on the other hand, offered the unique opportunity to investigate how photic input might relate to the season of birth data reported for birth years with the highest second quarter season-of-birth effect ever documented (O'Hare et al., 1980). As prior research has shown that rainfall 3 months before birth is significantly associated with the risk of becoming schizophrenic (Messias et al., 2001, 2006), this study focused on rainfall during the months encompassing what would have been the third trimester of gestation for births in the second quarter of the year.

## Materials and methods

### The former yugoslavia

**Figure 1:**
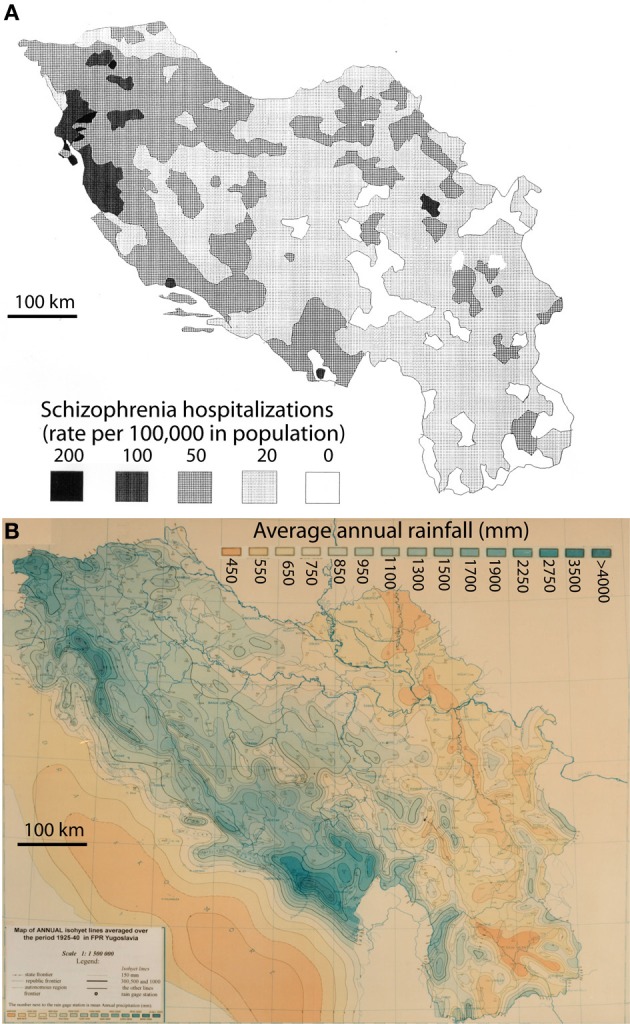
**Rates of schizophrenia hospitalization in the year 1931 (A) vs. the average annual rainfall, 1925–1940 (B) for the former Yugoslavia**. The hospitalization rates are plotted by place of the patient's birth (derived from Crocetti et al., 1964).

The hospitalization rates for schizophrenia by place of birth in Yugoslavia were derived from Crocetti et al. (1964). Those authors published a detailed map of the data put together by Kuljzenko (1933). During the course of one year (1931), hospitalization records had been obtained by Kuljzenko and co-workers for the whole of Yugoslavia and for each patient, the place of birth was noted. The scale of their plotted data was on the order of 100 sq. km. For the purposes of this study, the epidemiological map (Crocetti et al., 1964) was digitized by a draftsman using the program AutoCad (Figure 1, top panel) to enable digital overlay of epidemiological and meteorological data. The present study relied completely on the interpretation by Crocetti et al. (1964) of Kuljzenko's publication.

**Figure 2:**
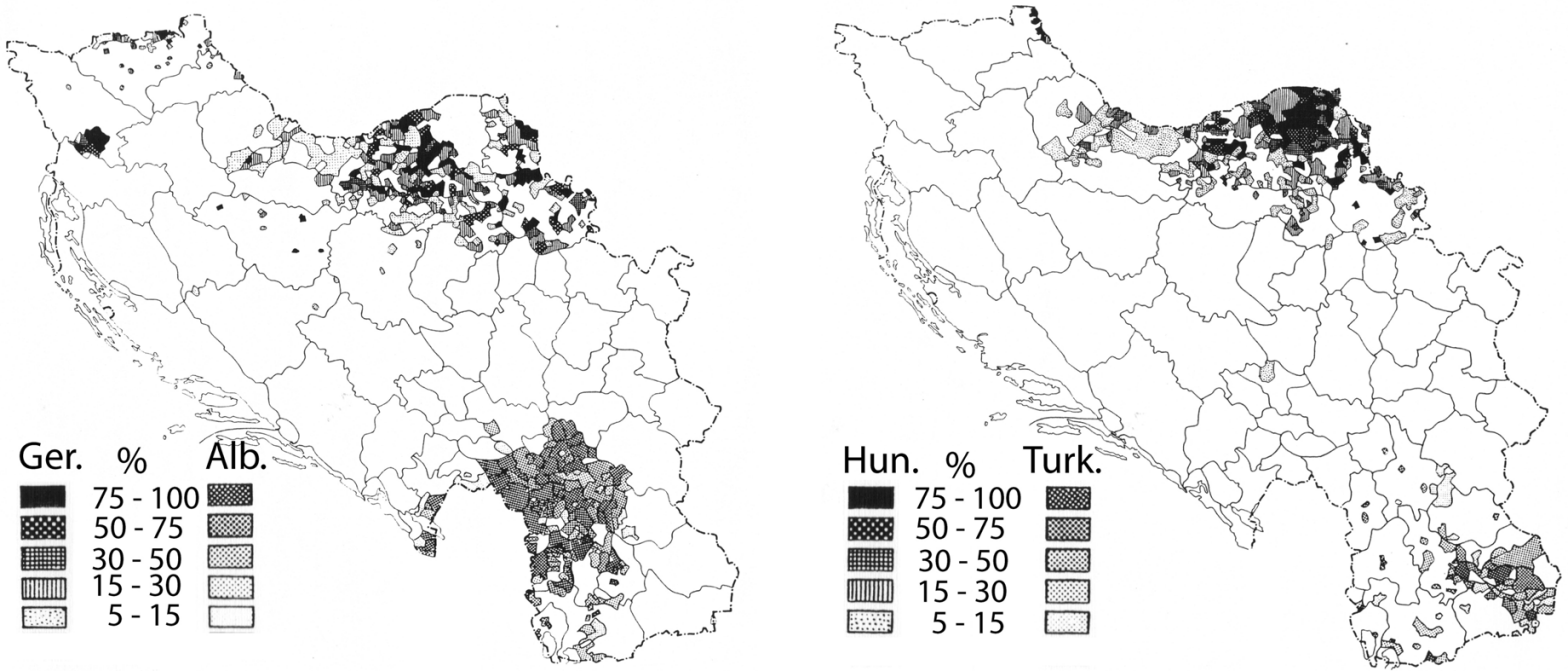
**Maps showing the distribution of non-Slavic ethnic groups within the former Yugoslavia (reprinted from Banac, 1984 with permission from Cornell University Press)**. The **left panel** depicts regions where Germans (Ger.) and Albanians (Alb.) constitute from 5 to 100% of the population. The **right panel** depicts regions where Hungarians (Hun.) and Turkish communities (Turk.) constitute 5–100% of the population. In the **left panel**, the regions with significant Germanic communities lie exclusively within the top half of the map, and the regions with significant Albanian communities within the bottom half of the map. In the **right panel**, the regions with significant Hungarian communities lie within the top half of the map and the regions with significant Turkish communities within the bottom half of the map.

The mean annual precipitation data (equivalent to rainfall) for Yugoslavia was provided by the Yugoslavian Hydrometeorological Institute (Figure 1, bottom panel). The data was for a 15 year period (1925–1940) encompassing the year of the hospital census, but not necessarily the year of birth of the patient. However, the decade-to-decade variation in rainfall is quite low (<7%; personal communication from the former Yugoslavian Federal Hydrometeorological Institute), and more importantly, the variation in rainfall totals is unlikely to have changed the pattern of rainfall in any significant way. Data was digitized for the author by the Yugoslavian Hydrometeorological Institute and the mean annual rainfall values were calculated for 0.5 degree cells, constituting roughly 2000 sq. km. The boundaries of each cell were then superimposed on the schizophrenia hospitalization map. For each level of hospitalization rate, the corresponding cells for rainfall were tallied by the author and mean values were calculated. Where the boundary of a mapped schizophrenia hospitalization rate excluded a portion of a rainfall cell, the rainfall data was weighted by the landmass for the proportion of the cell that was included. To exclude the potentially confounding effect of different ethnic populations, regions with non-Slavic ethnic groups that represented from 5 to ≤100% (Figure 2) were excluded from the final analysis.

### Ireland

The Irish Health Research Board publishes yearly compilations of mental health data, including first admission rates for schizophrenia by health board catchment area, and for 1991, a country-wide census was compiled for hospitalization at midnight on March 31, 1991. The period 1982–1991 (including the census year) was used by the author to calculate the mean first admission rates for each catchment area. The catchment area sizes were approximately 4600 sq km and up.

**Figure 3:**
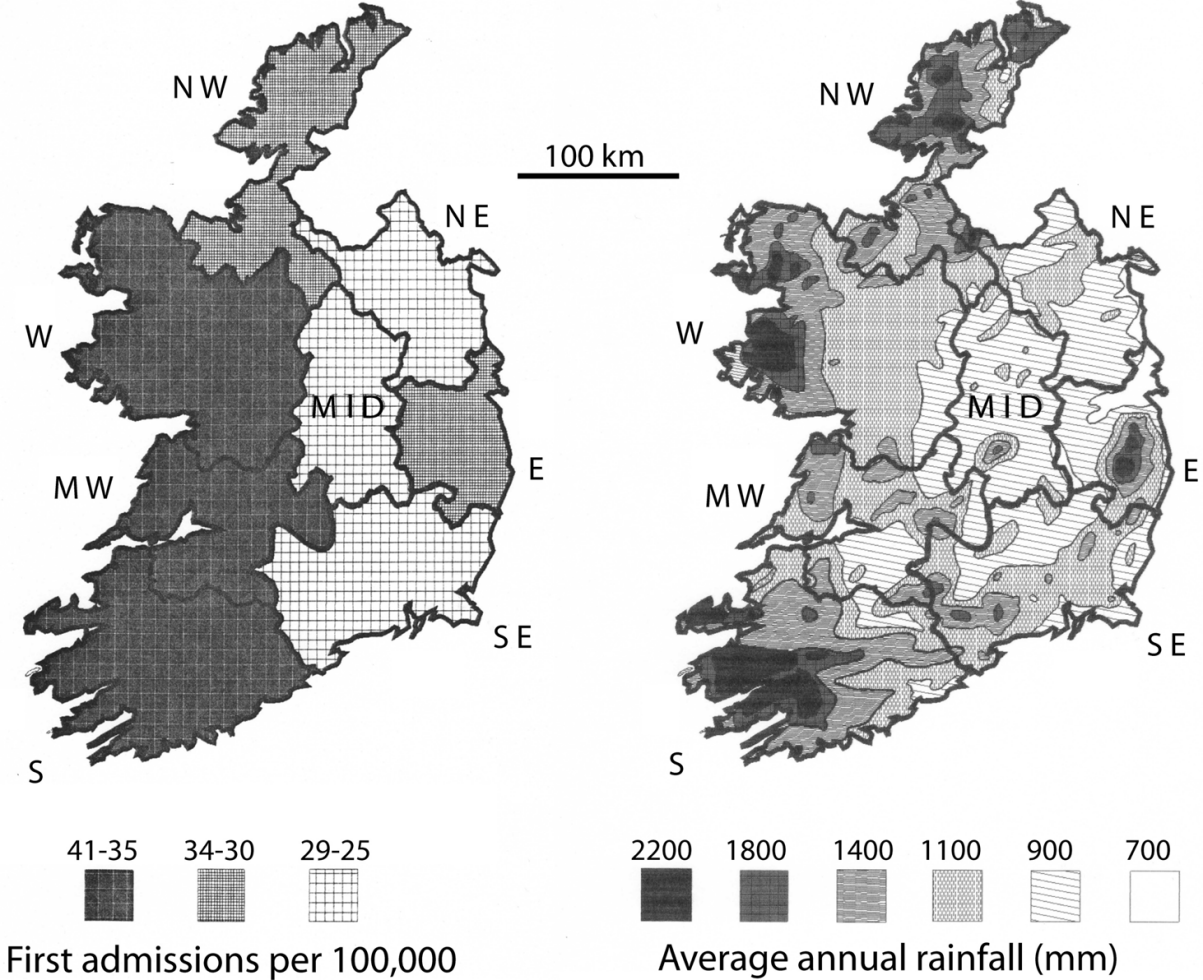
**Rates of schizophrenia 1st admissions (left panel) vs. annual rainfall (right panel) in Ireland**. The 1st admissions digital plot represent means of the yearly values in the outlined health catchment area (S, MW, W, NW, MID, NE, E, and SE), calculated for 1982–1991 from data published by the Health Research Board for Ireland (Activities of Irish Psychiatric Hospitals and Units Dublin, Ireland). The annual rainfall digital plot (derived from the Irish Meteorological Service, Galway and Logue, 1984) represents means of the yearly totals for 1941–1970, which would have included the birth years for many of the 1st admission patients.

Maps of the catchment areas for Ireland were digitized with the program AutoCad, encoded with the hospitalization rates and the 1st admission rates for schizophrenia (Figure 3, left panel). The catchment areas were then superimposed on a map of the mean annual rainfall for Ireland (Figure 3, right panel). The mean annual rain map was digitized by AutoCad from a map provided by J. J. Logue of the Irish Meteorological Service (Logue, 1984), representing rainfall data collected 1941–1970. Those years most probably included the year of birth of a good portion of the patient population creating the hospitalization and first admission rates used in this study. Since the isohyets of mean rainfall obviously did not match the boundaries of the maps for schizophrenia rates, the area covered by a given isohyet interval was digitally calculated. The percent of each catchment covered by a given isohyet interval was determined, and the mean rainfall for the region identified by summing the contribution of each area for a given isohyet interval, weighted by its percent contribution to the catchment area. The correction for population distribution (1986 census) was carried out by weighting the rainfall data by the population (as a percent of the total population in the particular catchment area) in the towns with 5,000 or more residents and assuming that the remaining land area exhibited a uniform population distribution.

**Figure 4:**
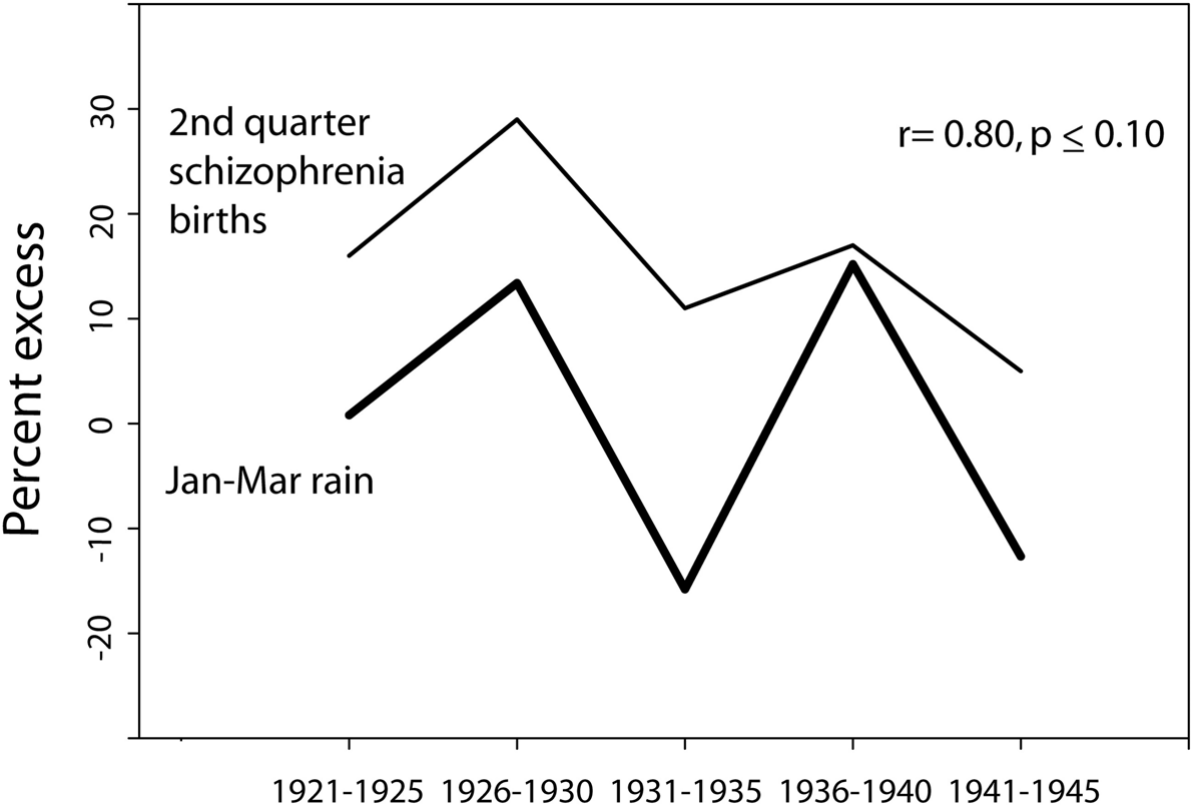
**The relationship between the percent excess (of average for weather stations in Ireland) of Jan–Mar rain and the percent excess of 2nd quarter schizophrenia births (as compared to the year-round quarterly average for 5 year period) for time periods spanning the years 1921–1945 (season-of-birth data derived from O'Hare et al., 1980)**. Note that Jan-Mar rain was not available from the series “British Rainfall” for the war year 1941, and thus the data point for the rainfall does not include 1941, though the schizophrenia data does include that year. To the extent that the weather in Ireland typically parallels the British Rainfall data mapped for Wales/West Midlands/Southwest England, the Jan–Mar rain would be expected to have been lower than average in 1941.

Season of birth data for schizophrenia for Ireland as a whole (not available per catchment area for different years) was obtained from O'Hare et al. (1980) for births in the years 1921–1955. The data was available in 5 year increments: 1921–1925, 1926–1930, etc. January through March (Jan–Mar) rain for those years was available only for the years 1921–1945 (with the exception of the war year 1941), obtained in map form as a percent excess of average, from the annual publication “British Rainfall.” The percent of the country covered by a given isohyet interval was calculated, and the correction for population distribution was carried out by weighting the rainfall data by the population (in this case, as a percent of the total population of Ireland) in the towns lying within a given isohyet interval (only for those with 5,000 or more residents) for the appropriate census year, and assuming that the remaining land area exhibited a uniform population distribution. For the birth years 1921–1930, the census data was derived from the 1926 Ireland census; for the birth years 1931–1940, the census data was derived from the 1936 Ireland census; and for the birth years 1942–1945, the census data was derived from the 1946 Ireland census. The relationship between the population-corrected rain data and the 2nd quarter season-of-birth data was then analyzed (Figure 4).

### Statistics

Where mean values were calculated for rainfall and for rates of schizophrenia, standard deviations are not reported because no group-wise comparisons are made of the means. The program Linear regression was carried out using the program SigmaStat to test for correlation between mean rates of schizophrenia and mean values of rainfall, and the resulting *r* value with the associated significance level (*p* value) is reported.

### Gene and peptide symbols

*Alpha-MSH* represents alpha-melanocyte-stimulating hormone.
*MC5R* represents melanocortin receptor-5 (for which alpha-MSH is an agonist).
*MCH* represents melanin-concentrating-hormone.
*MCHR1* represents melanin-concentrating-hormone receptor-1.
*MCHR2* represents melanin-concentrating-hormone receptor-2.

## Results

### The former yugoslavia

A 16 year period of mean rainfall values (1925–1940) was considered representative of the pattern of rainfall normally experienced in Yugoslavia during the late 19th and early 20th centuries, according to the Yugoslavian Federal Hydrometerological Institute. A visual comparison of the weather map for the former Yugoslavia (Figure 1A) and a map of hospitalization rates for schizophrenia in the year 1930 (Figure 1B; after Crocetti et al., 1964 and Kuljzenko, 1933), revealed a striking similarity in the patterns. To quantify this apparent relationship, the mean annual rainfall was calculated for regions experiencing a given rate of hospitalization (Table 1). The correlation between the hospitalization rate for schizophrenia by place of birth (Crocetti et al., 1964; after Kuljzenko, 1933) and mean annual rainfall was determined to be *r* = 0.96 (*p* = 0.008).

**Table 1:** **Rates of hospitalization and annual rainfall, Yugoslavia**.

**Hospitalized Schizoprenic Patients (per 100,000)^a^**	**Mean annual rain (mm)^b^**	**Mean annual rain (mm) wi diverse ethnic regions removed**
200	1780	1780
100	1470	1483
50	906	917
20	948	932
0	764	766

a Data obtained from Crocetti et al. (1964) for hospitalization rates in 1931.

b Data provided by the Yugoslavian Hydrometeorological Institute for the years 1925–1940.

Crocetti et al. (1964), confirmed the patterns revealed in Kulzjenko's work for Croatia only. However, apart from the methodology of the patient ascertainment, an additional concern in a country as diverse as Yugoslavia is the confounding effect that different ethnic groups would pose for a disease that is thought to be partly genetic in origin. The bulk of the former Yugoslavia was composed primarily of Southern Slavs (Banac, 1984) who were either Christian or Muslim in religion. Small pockets of German, Hungarian, Albanian, and Turkish communities populated Yugoslavia in 1921 (Figure 2, after Banac, 1984). The most direct way to assess the impact of these ethnic groups was to remove from the analysis those regions in which non-Slavs were a high percentage of the population (Table 1, right column). Doing so had no effect on the direction of the correlation or the significance level (*r* = 0.97; *p* = 0.008); thus, ethnic differences do not appear to be responsible for the variation in rates of schizophrenia within Yugoslavia.

### Ireland

The data for schizophrenia in Ireland (Table 2) was derived from yearly publications of the Irish (Health Research Board, 1972–1994), which provide a variety of hospital statistics including first admission rates and comprehensive censuses taken for point hospitalization rates.

**Table 2:** **Rates of hospitalization, first admissions and annual rainfall, Ireland**.

**Catchment area**	**Hospitalized Schizophrenic patients^a^ (per 100,000 gen. population)**	**1st Admissions for Schizophrenia (per 100,000 gen. population)**	**Mean annual rain^b^ (mm)**	**Mean annual rain weighted by pop. exposed^c^**
W	166.6	34.7	1274	1248
MW	122.4	40.7	1134	1134
S	124.4	37.5	1441	1360
NW	102.0	32.7	1378	1317
SE	99.0	29.4	1063	1013
MID	103.4	27.8	935	935
E	65.9	30.5	1045	933
NE	84.4	24.6	986	912

a Hospital census in 1991 (Health Research Board, Ireland).

b Mean annual rain in Ireland for the years 1941–1970.

c Population based on the 1986 census for Ireland.

The hospital census data presented are for the year 1991. Mean first admission rates were calculated for a 10 year period (1982–1991, inclusive), selected to include a comprehensive census year (1991) and selected as a time of relative stability in terms of the grouping of the hospital reporting system. The exception is 1991, when: (1) in the NE, Cavan and Monaghan began to report as a unit, (2) in the S, a new psychiatric unit was formed in Tralee (joint reporting with the hospital in Killarney) and (3) in the NW, a new psychiatric unit in Letterkenny began reporting with St. Conal's (also in the town of Letterkenny). The latter event may be responsible for the apparent jump in first admission rates for the NW at that time. However, it is also possible that the 1991 jump in NW first admission rates reflects compensation for under-reporting in the previous years, or is due to some other unknown factor.

For the number of patients hospitalized with schizophrenia, the correlation with mean annual rain is *r* = 0.52, *p* = 0.19. For first admissions of schizophrenics, the correlation with mean annual rain is *r* = 0.65, *p* = 0.084. Thus, there is trend toward a correlation between rainfall and 1st admission rates of schizophrenia, but the trend does not reach statistical significance. One difference between Ireland and Yugoslavia was that both the range of rates for schizophrenia and the range of rainfall values were smaller in Ireland, which decreases the power of the analysis. In addition, the scale of the data maps was more detailed for Yugoslavia than for Ireland. Thus, small scale variations in rainfall affecting a non-uniform distribution of population were more likely to confound the Irish data. For example, in County Donegal, the mean rainfall is 1455 mm, but relatively small proportion of the population lives in regions with rainfall values of that magnitude.

Thus, to more accurately represent the amount of rainfall the average person in a catchment area experiences, the rain data was weighted by population distribution (Table 2; Materials and Methods). This analysis assumed a similar distribution of population existed during the time period when rainfall might have exerted an effect. The results show that if the rain data is weighted by the exposure of the most populous cities in a catchment area (Table 2), the 1st admissions for schizophrenia correlated significantly with mean annual rainfall (*r* = 0.71, *p* = 0.047), and hospitalization rates showed a trend to correlate with mean annual rainfall (*r* = 0.65, *p* = 0.082).

### Season of birth effect in ireland

In 1980, O'Hare et al. published a study tracking the season of birth effect for births over a 35 year period in Ireland (1921–1955), reported for 5-year intervals. When compared to the expected number of births of individuals who would go on to develop schizophrenia, based on the total number of births in each quarter, the spring quarter showed a marked 29% excess of future schizophrenics born in the 5 year period of time 1926–1930. The rainfall data available for that time period also showed some notable trends. To quote from the publication British Rainfall (which covered Ireland during those years), “1928 was the 6th successive year in which the rainfall over the British Isles as a whole was in excess of the average… we have to go back to the ‘seventies to find so long a run of wet years…. A run of six consecutive years each with an appreciable excess is, however, unprecedented.”

Of the years encompassed by the season of birth study, maps of rain data for Ireland were available from the British Meteorological Service spanning the years 1921–1945, with the exception of 1941. The mapped isohyet lines made possible the easy calculation of the surface area covered by particular rainfall patterns. Figure 4 illustrates the similarity in patterns between the national Jan–Mar quarterly excess in season of birth for schizophrenia in those years and the national excess or deficit in January to March rain for those years. When the annual rain data was weighted by the underlying population distribution affected by each particular rain pattern, the degree of correlation with season of birth effect was high (*r* = 0.80) and trended toward significance (*p* ≤ 0.10).

The possibility of a correlation was explored between 1st admissions for schizophrenia (Table 2) and degree of season-of-birth effect as reported by O'Hare et al. (1980), but no correlation was found (*r* = 0.0034, *p* = 0.994).

## Discussion

The data assembled and analyzed for this study are entirely consistent with a role for photic cues in human development and behavior, in this case behaviors as pronounced as those seen in schizophrenia. The observed patterns could reflect differences in genetic traits of the populations, gene-environment interactions specific for certain genotypes in certain environments, and/or phenotypic plasticity that can occur for all genotypes.

The season-of-birth pattern strongly supports work by Messias et al. (2001, 2006) who demonstrated a remarkably similar finding for schizophrenia season-of-birth in Brazil, where the main variation in seasonal weather is limited to a January through March rainy season. In those studies, a significant association was found between rainfall during a given month and the number of individuals with schizophrenia with birth dates 3 months later. Similarly, McGrath et al. (2002) found a significant association between variations in perinatal sunshine duration and the season of birth effect in schizophrenia. Furthermore, a recent report demonstrates an equivalently strong season-of-birth effect in multiple sclerosis with peaks in April and May and in parallel with schizophrenia (Torrey et al., 1997; Davies et al., 2003), deficits in births of future multiple sclerosis patients during October and November (Dobson et al., 2013), a finding the authors attribute to variations in sunlight during gestation and to the resulting variations in vitamin D availability.

Although the association between schizophrenia and the influence of heavy rainfall on photic input represents a potentially important avenue of research, this study outcome does not preclude the involvement of other environmental variables influenced by rainfall. The critical variable that rainfall represents could also include lower temperature (although rainfall does not always correspond to lower temperature) and infectious disease (spread more easily when people must spend more time indoors). Others have shown (Hare and Moran, 1981; Kinney et al., 1993) that the degree of the season of birth effect for schizophrenia was proportional to the severity of weather near birth, but in that case an association was found with cold temperatures during the last trimester. Similarly, Kendell and Adams found an association with low temperature 6 months prior to birth (1991) and Gupta and Murray (1992) report an association between environmental temperature and the incidence and outcome of schizophrenia. Data presented here for Ireland would argue against infections as underlying the association between rainfall and schizophrenia because the rates for 1st admissions are highest in the rural south and west, where infectious disease spread through crowded indoor quarters was less likely than in the eastern urban areas. Furthermore, an aspect of the season-of-birth effect that has been somewhat overlooked (Torrey et al., 1997) is the consistent deficit in schizophrenic births occurring in the late summer and early fall, particularly evident at higher latitudes (Davies et al., 2003). The excess/deficit finding is more compatible with cyclic, seasonal decreases and increases in light than with spread of any single infectious agent. Despite the fact that peaks in specific infectious diseases certainly do occur for particular months, those peak months are not usually matched by a large deficit in a couple of months at the opposite end of the year.

### The processing of photic stimuli in animals

The availability of sunlight is undoubtedly one of the most important environmental factors that influence survival. Animal physiology is accordingly geared to respond to changes in both the sunlight intensity and duration, i.e., photoperiod. Photoperiod is defined as the length of time a given species perceives photic stimuli during the day and is obviously specific to the season of the year and to latitude. The response of the pineal to changes in the photoperiod involves regulation of melatonin production, a hormone integrally involved in setting the circadian clock (Bartness and Goldman, 1989). From the survival standpoint, a change in photoperiod is more informative for long term conditions in the postnatal environment than is temperature and for most animals, the photoperiod determines whether reproduction occurs or not. In species that can reproduce at different times of the year or year round, the effects are more subtle and relate to postnatal hormonal levels, circadian entrainment and somatic measures of development.

Photoperiod is sensed via projections along the retino-hypothalamic pathway to the pineal gland, operating as a step function (Prendergast and Zucker, 2012). Below a given intensity range, there is no response and above that range, the response is constant until the light level drops again. Suppression of melatonin synthesis in the pineal progresses in the early dawn with the first faint signal that the sun will be rising soon. The sensitivity of response is species specific, such that 1 lux is reported to be sufficient to significantly suppress melatonin synthesis in the Syrian hamster (Brainard et al., 1982). But at 119 lux, a level comparable to a clear summer sunrise at northern temperate latitudes (Didrikas and Hansson, 2009), the human pineal will generally have downregulated melatonin production by only 50% (Zeitzer et al., 2000) with full suppression by ~2500 lux (Coetzee et al., 1989; Arendt, 1998).

The relevant cues provided by photoperiod can be delivered both pre-and postnatally (e.g., voles, Lee and Zucker, 1988; collared lemmings, Nagy et al., 1993; Siberian hamsters, Stetson et al., 1986; Shaw and Goldman, 1995; and Prendergast et al., 1996; sheep, Ebling et al., 1989; and red deer, Adam et al., 1992). Perhaps of greatest importance to the behavior of interest, postnatal dopaminergic tone is influenced by the photoperiod experienced *in utero*. Dopamine controls prolactin levels, the most obvious expression of which is coat thickness and/or color in animals (Hoffman, 1978; Lee and Zucker, 1988). A short photoperiod upregulates hypothalamic dopaminergic activity, inhibiting prolactin release and initiating the development of a winter coat. Postnatal administration of dopaminergic antagonists can block development of the winter coat, whereas dopaminergic agonists promote a winter coat (Badura and Goldman, 1992; Gower et al., 1993). To what extent such striking gene-environment interactions are controlled by epigenetic changes is not known for mammals, though epigenetic modifications in response to photoperiod have been well documented in plants (Kim and Sung, 2010). Many photoperiod effects controlled by the pineal are encoded by the peptide hormone *alpha-MSH* (Kastin et al., 1967a,b), which is upregulated in response to long photoperiods, predominantly expressed in the cells of the intermediate pituitary and centrally, in the arcuate nucleus of the hypothalamus (O'Donohue and Dorsa, 1982; Hadley, 1984; Khachaturian et al., 1985).

The timing of photoperiod effects relevant to behavior could theoretically include events as early as the time of conception. As proposed by Jongbloet (1975) and Pallast et al. (1994), increased light duration during the summer could lead to release of ova that are over-mature and predisposed to defective development. However, the pre-natal critical period for the major photic effects on animal behavior and development probably lies closer to the equivalent of the last trimester in humans (Hoffman, 1978; Reppert, 1985; Stetson et al., 1986; Weaver et al., 1987; Lee and Zucker, 1988; Nagy et al., 1993; Bellavía et al., 2006; Butler et al., 2007). The types of behaviors influenced by photoperiod are species and gender dependent, and include the more bold behaviors observed for female Brazilian guinea pigs born in spring, whereas males do not show such clear differences (Guenther and Trillmich, 2013). Other behavioral effects are induced by a short postnatal photoperiod and include elevated measures of anxiety and depression seen in adult Siberian hamsters, collared lemmings, and nocturnal rodents, as well as reductions in learning and memory capacity seen in male white-footed mice (as reviewed by Walton et al., 2011).

In addition to photoperiod response, there are responses to sunlight intensity, some of which are not mediated by the pineal. In contrast to most other animals, humans have a large cutaneous surface area that is responds to sunlight by proportional (not step-wise) adjustments to sunlight intensity for both vitamin D (Chen et al., 2007) and melanotropin production (Farooqui et al., 1993; Chakraborty et al., 1996; Hiramoto et al., 2003).

Although sunlight-induced vitamin D is not an important source of vitamin D for lower animals, it has been shown that prenatal dietary vitamin D in Sprague-Dawley rats has significant effects on postnatal anxiety and social behaviors (Pan et al., 2013).

Therefore, at issue is which of these photic response processes might relate to observations that rates of schizophrenia vary with latitude, season of birth and rainfall? Photoperiod could certainly underlie a phenotypic response to latitude and season, but unlike processes modulated by sunlight intensity, it is not affected by weather. Rather, entraining the photoperiod is strongly tied to the calendar date and is seemingly independent of year to year fluctuations in precipitation or cloud cover. One of the most informative observational studies in this regard involved the coat color change in the snowshoe hare, in which it was demonstrated that a year with an unusually heavy spring snowfall pattern (and hence, cloud cover) did not change the date at which coat color changed from white to brown, leaving some animals brown against a white background (Mills et al., 2013).

Although weather does not alter photoperiod entrainment by the pineal, there may nevertheless be neurophysiological consequences resulting from weather changing the rate and the degree to which pineal melatonin is suppressed during the daylight hours. Thus, even the snowshoe hare study described above revealed a possible role for sunlight intensity, in that the hares began the development of a brown coat at the correct calendar time in a snowy spring but completed the transition from white to brown at a slower rate than during a less snowy spring (Mills et al., 2013). A pineal-mediated effect exerted by low light intensity occurring during a relatively long photoperiod has also been directly examined in birds, for which Kumar et al. (2007) found a delay in reproduction, explaining why in wild bird populations, heavy rainfall can similarly delay reproduction (reviewed by Small and Moore, 2009). The magnitude of the impact of a heavy rainy season on sunlight intensity has been quantified for the Tibetan plateau, where consistently heavy rain in summer decreases both the daylight duration and the sunrise to sunset light levels equivalent to those seen in spring (Liu et al., 2012). For humans, the effect of rain on photic input is further complicated by the need to be indoors during heavy rain and for much of the time period covered by the present study, the populations in question would have had limited alternatives for indoor lighting. Even with optimal “daylighting” strategies seen now in modern building design, the maximum indoor sunlight levels achieved midday on a clear Stockholm day in December, for example, barely reaches above 200 lux (De Carli and Valeria De Giuli, 2009), with values less than ~50 lux for at least some of the working hours after sunrise but before sunset. Cloud cover and rainfall could be expected to further decrease those levels by 70 and 83%, respectively (Luccini et al., 2003), depending on the thickness of cloud cover and the intensity of rain (Calbo et al., 2005).

With respect to the possible role of sunlight intensity in behavioral phenotypes, it has been proposed that cutaneous vitamin D generation may be involved in gestational effects that modulate the eventual development of schizophrenia (McGrath and Welham, 1999). In addition, other hormones of interest are produced in the skin in response to the intensity of the natural spectrum, including the melanotropin *alpha-MSH* (Farooqui et al., 1993; Lin and Fisher, 2007). Any matching CNS elevations of *alpha-MSH* via cues from the pineal would be expected to have important effects on learning and memory (LaHoste et al., 1980; Beckwith et al., 1989; Machado et al., 2010; Shen et al., 2013), the processing of sensory information (Miller et al., 1993) and feeding behaviors (Nahon, 1994, 2006).

### Phenotypic plasticity, genetic traits and gene-environment interactions

The season-of-birth effect is clearly an example of phenotypic plasticity but it also offers a window into forces that may have selected for genetic change. There are many examples in animal evolutionary history of phenotypic plasticity giving way to related “hardwired” traits (Van Buskirk et al., 1997), a phenomenon that some evolutionary biologists term the “flexible stem hypothesis” (Wund et al., 2008; Tebbich et al., 2010; Muschick et al., 2011). The physiology of light-responsive genes normally seen with seasonal environmental changes in light levels would also be engaged when year round light levels become different, as happens for individuals migrating from southern to northern climates. But over evolutionary time, the more completely adapted physiologies will exhibit permanent genetic traits that have been selected for by the new environment. A potentially relevant example would be the phenotypic plasticity identified in monozygotic twins discordant for bipolar disorder, who carry epigenetic methylation differences in the receptor for the functional antagonist of *alpha-MSH*, the melanotropin receptor known as *GPR24* or *MCHR1* (Dempster et al., 2011). Yet, hardwired differences in that gene were also selected for and have been found to be associated with bipolar disorder across unrelated individuals (Miller et al., 2009).

The need for vitamin D may have played an important role in selecting for polymorphisms in a variety of light-responsive genes, including the melanotropins. Any genetic polymorphism that enhances the ability of vitamin D to be generated from light would have been advantageous in low-light regimes, except when vitamin D was easily obtained from the diet. It is well known that a lack of vitamin D causes rickets, which would have had a negative impact the ability to perform the physical work necessary to survive in historical times, but more importantly, frequently caused fatal outcomes during delivery because of the improper configuration of pelvis in severe rickets (Harrison, 1966; Cruickshank, 1967; Konje and Ladipo, 2000). Genetic polymorphisms that increase risk of fatal outcomes prior to successful reproduction are under intense negative selective pressure, readily apparent within a few generations (reviewed by Miller, 2009). In such a manner, certain polymorphisms in melanotropin genes may have become more prevalent in low-light environments if they positively affected the natural synthesis of vitamin D by reducing the synthesis and sequestration of melanin (Valverde et al., 1995). The evolutionary trade-off in this case would have been an increased prevalence of polymorphisms in melanotropin genes (*MCHR1, MC5R, MCHR2*) of risk for schizophrenia (Severinsen et al., 2006; Miller et al., 2009; Demontis et al., 2012).

Although no genetic associations between vitamin-D receptors or enzymes involved in its formation or degradation have yet been identified for schizophrenia, that outcome does not necessarily mean that vitamin D is without effect in modifying the phenotypic plasticity that is obviously present in the disease. An interaction between vitamin D and the melanotropin system during development has been demonstrated by Eyles et al. (2007) who found that prenatal vitamin D deficiency in rodents leads to elevations in the functional antagonist of *alpha-MSH*, the melanotropic peptide *MCH*. However, McGrath and colleagues have also shown that the relationship between vitamin D in gestation and subsequent schizophrenia may be complex, in that those with low maternal vitamin D are at increased risk of bearing offspring who become schizophrenic as are those with overly high vitamin D (McGrath et al., 2010a).

What might be the relative impact of phenotypic plasticity vs. genetic traits of risk? For schizophrenia, the calculations show that the impact of the season-of-birth effect is not minor but rather roughly equivalent to that of family history of disease (Mortensen et al., 1999). For the northern hemisphere, the population attributable risk caused by a late winter/spring birth is on average 3.3% (Davies et al., 2003) but ranges to 10.5% in some locales (Mortensen et al., 1999), whereas the population-attributable risk if a parent or sibling was schizophrenic was 5.5% in the Mortensen et al. study (1999).

The season-of-birth effect could also be viewed as an example of gene-environment interaction because the effect is not uniform across the population; rather, it is reported to be greatest in those without a family history of the disease (O'Callaghan et al., 1991). Similarly, gene-environment interactions may underlie the remarkably increased risk of schizophrenia for immigrant populations from Afro-Caribbean countries who have relocated to the U.K. (McGovern and Cope, 1987; Wessely et al., 1991; Harrison et al., 1997; Sharpley et al., 2001; Coid et al., 2008), as compared to the incidence of schizophrenia in their native lands (Hickling and Rodgers-Johnson, 1995; Bhugra et al., 1996; Mahy et al., 1999) and as compared to immigrants from other countries (Coid et al., 2008). Barring the unlikely possibility of preferential migration of the most genetically at-risk individuals, their increased predisposition to schizophrenia in the U.K. must be triggered by some factor in the environment interacting with particular aspects of their genetic background. The model put forth in this paper would presume that the culpable environmental factor is related to lower light levels in northerly climates, although the effect of the stress of immigrating to a different culture cannot easily be discounted, as discussed by Coid et al. (2008). In addition, rates of usage of illicit drugs may be higher amongst Afro-Caribbeans immigrants, particularly of concern if the drug of choice is cannabis (McGuire et al., 1995; Moore et al., 2007; Arendt et al., 2008; Di Forti et al., 2009; McGrath et al., 2010b). The rate of cannabis use in the Afro-Caribbean immigrant population may be somewhat higher vs. long term residents of the U.K. (Harvey et al., 1990) but may not be higher than rates in their home countries which have been reported to be already quite high (reviewed by Sugarman and Craufurd, 1994; Maharajh and Konings, 2005). However, other investigators found no elevation in drug use in Afro-Caribbeans in the U.K. as compared to non-immigrants (Cochrane and Bal, 1989; McGuire et al., 1995; reviewed by Coid et al., 2008).

Obviously, two of the major light-responsive physiological systems that would be strongly affected in Afro-Caribbean immigrants to the U.K. would be UV-induced vitamin D and the melanotropins. UV-induced vitamin-D would be expected to be particularly low for these individuals (Ford et al., 2006; Chen et al., 2007), as would the stimulation of the light-responsive melanotropin system in the climatic regime found in the U.K., since skin pigment would be expected to lessen the responsiveness of *alpha-MSH* levels to the relatively low level of UV radiation found there (Holzmann et al., 1983; Altmeyer et al., 1986; Chakraborty et al., 1996). It is noteworthy that the schizophrenia risk is higher for the second generation than the first (McGovern and Cope, 1987; Coid et al., 2008), suggestive of epigenetic effects during growth and development.

There is reason to believe that gene-environment interactions may also underlie certain of the genetic associations with schizophrenia identified in the melanotropin genes (Severinsen et al., 2006; Miller et al., 2009; Demontis et al., 2012). When evaluating genetic association studies, it must be kept in mind that they occur in particular environments. Thus, the resulting associations can be for genetic polymorphisms which exhibit strong interactions with that environment as well as those that don't. Based on the “flexible stem” hypothesis, the expectation would be that if an association was found for an ancestral polymorphism of relatively lower prevalence in the study environment than in the ancestral environment, this outcome might be indicative of a gene-environment interaction causing disease in the study environment. The ancestral polymorphism would represent the “flexible stem” form of the gene, a form which eventually was selected against. Such may be the case for the association between schizophrenia and a coding change in the *MC5R* gene identified in a temperate-zone genetic association study (Miller et al., 2009). Against a background of other risk genes (*TDO2* and *MCHR2*), the *MC5R* polymorphism of risk (rs2236700) was unexpectedly found to be the ancestral allele, an allele roughly twice as prevalent in the Yoruba peoples of Nigeria as in Caucasians represented by the CEPH collection (HapMap, www.hapmap.org). Based on the fact that meta-analyses showed a higher incidence of schizophrenia with higher latitude (Saha et al., 2006), it would not be expected that an allele more prevalent in Nigeria would be associated with a greater risk of disease. But the key fact to remember is that in the genetic association study of interest (Miller et al., 2009) the disease was diagnosed in people living in another climate, i.e., in temperate zone latitudes.

Thus, the association of MC5R with schizophrenia could theoretically represent a gene-environment interaction relevant to the outcome seen for Afro-Caribbean peoples migrating to the U.K. *MC5R* is a receptor for *alpha-MSH* which, as described above, is one of the light-responsive hormones elevated during long-day photoperiods and when light levels are more intense. The function of its *MC5R* receptor is diverse, ranging from stimulation of sebaceous glands (Eisinger et al., 2011) to immunoregulation (Taherzadeh et al., 1999; Taylor and Namba, 2001), to behavioral effects that include modulation of aggression (Morgan et al., 2004). Because *MC5R*'s association with schizophrenia was identified against the genetic background of a risk allele for the immunomodulatory kynurenine pathway enzyme *TDO2* (Miller et al., 2009), it is most likely that the key action in this case would be the reported inhibition of *IFN*γ expression by *MC5R* (Taylor and Namba, 2001). *IFN*γ stimulates the expression of IDO (Taylor and Feng, 1991), one of the other enzymes responsible for activating the immunomodulatory, and pigment-generating kynurenine pathway. Kynurenine pathway activation has been demonstrated in several studies of schizophrenia (reviewed by Schwarcz et al., 2012). Although the necessary studies have not yet been done to determine the functional effect of the risk allele of *MC5R*, if it were to be the case that it coded for a less sensitive version of the *MC5R* receptor, the result would be increased activation of the kynurenine pathway, further augmenting pathway flux in low light environments where the *MC5R* agonist *alpha-MSH* is already low.

### Other evidence for the involvement of the physiology of photic response in the expression of schizophrenia

Additional evidence for light-responsive melanotropin involvement can be found in an alternative mechanism of action proposed for antipsychotic drugs (Miller, 2013), based on the observed reaction between antipsychotic drugs and a neurotoxic catecholamine breakdown product to form the more innocuous pigment polymer, melanin. Consistent with this outcome, the melanotropin *alpha-MSH*, which enhances melanin formation and its sequestration, has been shown to normalize sensory gating in an auditory model of a schizophrenia endophenotype (Miller et al., 1993). In contrast, the melanotropin that inhibits the formation of melanin and its subsequent sequestration (*MCH*), inhibits effective sensory gating (Miller et al., 1993; Chung et al., 2011). Furthermore, nutritional imbalances that perturb melanogenesis can also elicit symptoms of psychosis (reviewed by Miller, 2011).

## Conclusions

The correlations between epidemiological data and light levels are strong for schizophrenia, and should not be ignored in the search for means of lowering the incidence of this major mental disorder. The need for vitamin D may have affected not only gene frequencies of relevance to schizophrenia but may also have modulated gene-environment interactions that can occur in differing light regimes. We modern humans tend to downplay the effect of environment in controlling our health and well-being, particularly in regards to an environmental force such as light that can be replaced by an artificial source. Yet it would be unwise to discount the importance of natural light, particularly when our reliance on it was so high during our recent evolutionary past.

## Limitations of the study

The methods of ascertainment of cases in the data set for the former Yugoslavia cannot be effectively validated. Much criticism of diagnostic methodology has been directed toward many studies of schizophrenia, and the current study is particularly vulnerable to such critiques. Despite the fact that the methods employed in Ireland have been overseen and well-supervised by the Health Research Board of Ireland, their methodology undoubtedly changed over time. Furthermore, no correction for potentially confounding variables such as demographics of the local population, drug use or obstetrical complications was possible.

### Conflict of interest statement

The author declares that the research was conducted in the absence of any commercial or financial relationships that could be construed as a potential conflict of interest.
